# Coordinate Representations for Interference Reduction in Motor Learning

**DOI:** 10.1371/journal.pone.0129388

**Published:** 2015-06-12

**Authors:** Sang-Hoon Yeo, Daniel M. Wolpert, David W. Franklin

**Affiliations:** 1 Computational and Biological Learning Lab, Department of Engineering, University of Cambridge, Cambridge, United Kingdom; 2 School of Sport, Exercise and Rehabilitation Sciences, University of Birmingham, Edgbaston, Birmingham, United Kingdom; University of California, Merced, UNITED STATES

## Abstract

When opposing force fields are presented alternately or randomly across trials for identical reaching movements, subjects learn neither force field, a behavior termed ‘interference’. Studies have shown that a small difference in the endpoint posture of the limb reduces this interference. However, any difference in the limb’s endpoint location typically changes the hand position, joint angles and the hand orientation making it ambiguous as to which of these changes underlies the ability to learn dynamics that normally interfere. Here we examine the extent to which each of these three possible coordinate systems—Cartesian hand position, shoulder and elbow joint angles, or hand orientation—underlies the reduction in interference. Subjects performed goal-directed reaching movements in five different limb configurations designed so that different pairs of these configurations involved a change in only one coordinate system. By specifically assigning clockwise and counter-clockwise force fields to the configurations we could create three different conditions in which the direction of the force field could only be uniquely distinguished in one of the three coordinate systems. We examined the ability to learn the two fields based on each of the coordinate systems. The largest reduction of interference was observed when the field direction was linked to the hand orientation with smaller reductions in the other two conditions. This result demonstrates that the strongest reduction in interference occurred with changes in the hand orientation, suggesting that hand orientation may have a privileged role in reducing motor interference for changes in the endpoint posture of the limb.

## Introduction

Storing and retrieving various motor skills is an essential function of our sensorimotor control system. Previous studies have shown that contextual cues, sensory signals that are not directly related to the actual control of the movement, but that contain information about the dynamics, play an important role in formation and recall of various motor memories. Experimental studies investigating the role of contextual cues have primarily focused on the inference occurring in reaching movement under opposing force fields. While subjects can learn each force field in separate sessions [1,2], their ability to learn is substantially impoverished when these two force fields are presented alternately or randomly in a single session. This is due to the interference that occurs when our sensorimotor control system tries to encode two motor memories but is unable to distinguish them [3–8]. This interference can sometimes be reduced when subjects are provided with a contextual cue indicating the type of the upcoming force fields [9–15]. The efficacy of a given contextual cue can be quantified by measuring the reduction in interference that it causes [11].

Previous studies have shown that a difference in endpoint posture of the limb, which is the main focus of this paper, provides a particularly strong contextual cue that can reduce interference [5,11,16–18]. Although these studies show that learning at one location does not interfere with learning at another location, as long as there is a sufficient distance between the two, an important question is what is the key metric of the spatial separation. In other words, there is ambiguity as to whether the reduction in interference is due to the change in hand location in Cartesian space, changes in the joint angles in intrinsic space, or changes in the hand orientation. In particular, studies of both grasping [5,19] and reaching [20] have suggested that the orientation of hand, or tool within the hand, could play a crucial role in motor learning.

The goal of the current study is to investigate the reduction in interference as a function of spatial separation in three coordinate representations: 1) Cartesian hand position, 2) shoulder and elbow joints angles, and 3) hand orientation in extrinsic space. We define the hand orientation in extrinsic space (rather than just by the wrist angle) to reflect the way in which the dynamics of a hand-held tool change in extrinsic space when the configuration of the arm changes [20]. Therefore, hand orientation has a special meaning separate from the shoulder, elbow and wrist angles. This is because, when holding a tool in the hand, its dynamics in Cartesian space depends on the hand orientation independent from the set of three joint angles. For example, there are many combinations of the three angles which have the same hand orientation and therefore do not change the orientation of a hand held tool in space. Therefore, we consider hand orientation as synonymous with the orientation of a tool and hence consider it as separate from the other two coordinate systems. In addition, the control of the wrist is fundamentally different from that of the shoulder and elbow joint in that is has far greater stiffness during reaching. Indeed, the wrist angle shows no perturbation or aftereffect when reaching under novel dynamic environments (see for example [20]).

**Fig 1:**
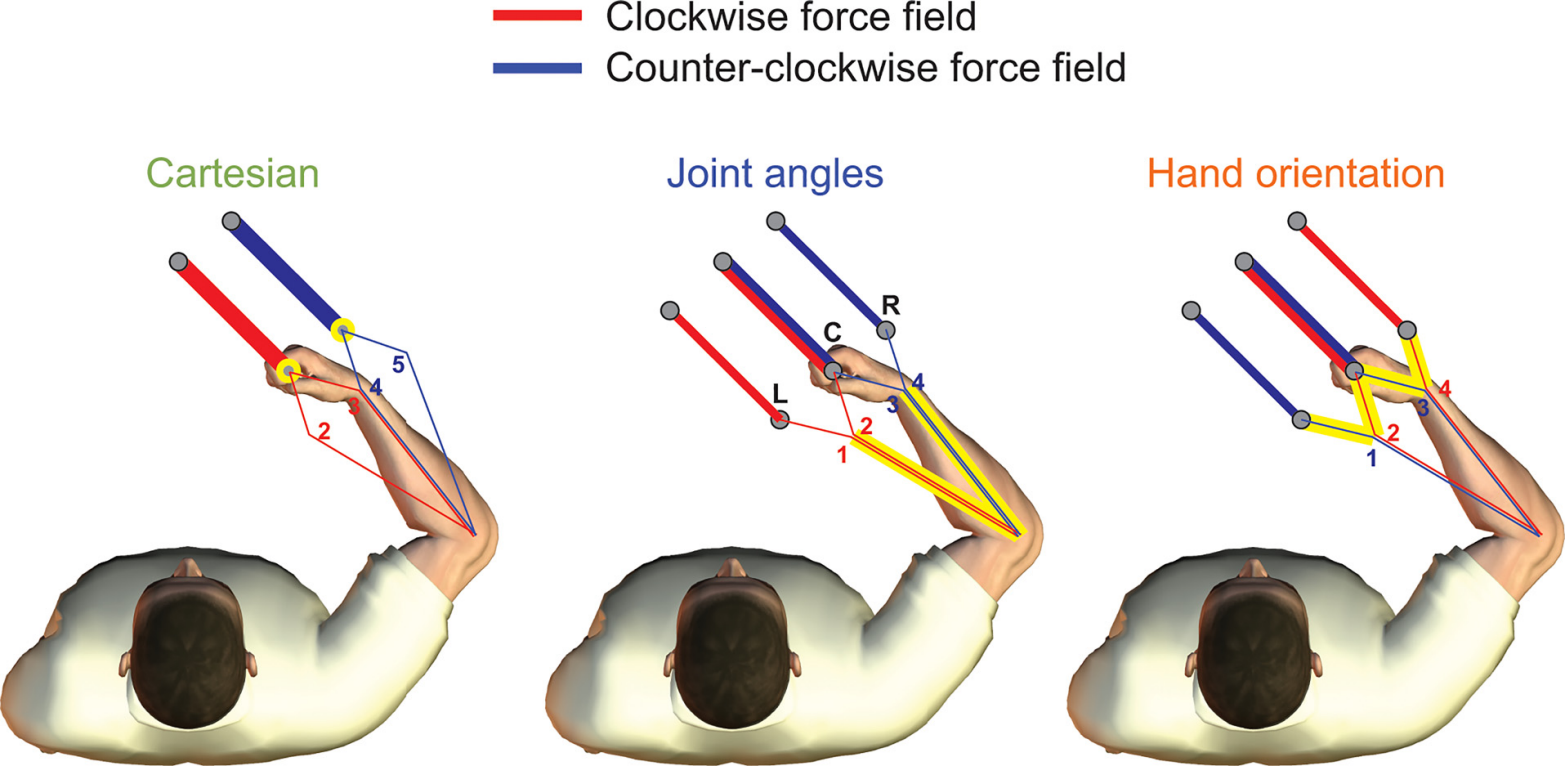
Experimental design. Five different limb postures were defined (numbered 1 to 5) which varied in their endpoint hand location, hand orientation and shoulder and elbow joint angles. Each group of subjects only experienced 4 of the postures and in the exposure epoch of the experiments a clockwise force field (red) was applied to movement made in two of the postures while a counter-clockwise force field (blue) was applied for movements made in the other two postures. In the Cartesian condition, these two opposing force fields were only uniquely separated in the hand location specified in Cartesian space (see yellow circles). In the Joint angle condition, the opposing force fields were only uniquely separated in the joint angle space of the shoulder and elbow (yellow lines). Finally in the hand orientation condition, the opposing force fields were uniquely separated based on the hand orientation. Force field directions for each trajectory were counterbalanced across the subjects.

To avoid the ambiguities of previous studies, our experiment was designed to separate the contributions of these three representations, so that the contextual effect of each coordinate system could be tested without being affected by the other two. We used three conditions (Fig 1), each of which is designed to test the effect of just one of three coordinate representations. For each condition, clockwise (CW) and counter-clockwise (CCW) velocity-dependent curl force fields were assigned to four limb configurations in such a way that the force field direction is uniquely specified by changes in only one coordinate representation, but cannot be determined based on changes in either of the other two. Therefore, we consider whether discrete settings of the hand orientation, Cartesian location of the hand or joint angles allows learning of opposing force fields. Such discrete settings can be considered as contextual cues to the direction of the force field where these contextual cues represent changes in only one of the coordinate representations. This allowed us to examine individually the effect of each of the three coordinate representations in turn. If subjects are able to reduce the interference in a certain condition, it indicates that changes in the corresponding coordinate representation can be used to separate motor memories.

## Materials and Methods

Twenty-four naive subjects (12 males) participated in the experiment (mean age ± sd, 26.8 ± 5.1 years). All subjects were right handed according to the Edinburgh handedness inventory [21] with no reported neurological disorders.

### Ethics Statement

The Cambridge Psychology Research Ethics Committee approved the study, and subjects gave written informed consent before participating.

### Experimental Apparatus

Subjects were seated, and grasped the handle of a vBOT planar robotic manipulandum [22] with their right hand. Position and orientation of the handle were measured at 1kHz using optical encoders and the computed forces were applied to the handle using torque motors. The endpoint forces between subject and the manipulandum were measured using a 6-axis force-torque transducer (ATI Nano 25, Industrial Automation, NC, USA) at 1 kHz. In addition, a goniometer (S700 Joint Angle SHAPE SENSOR, Measurand Inc.) was used to measure the subjects’ elbow angle, sampled at 1 kHz. The goniometer was attached to the lateral side of the subject’s right arm across the elbow, using velcro-straps.

Visual feedback was provided using a computer monitor mounted horizontally above the vBOT and the monitor image was projected into the plane of movement via a mirror. Subjects were prevented from viewing their hand directly, and the virtual reality system was used to overlay images into the plane of movement, updated at 60 Hz.

The basic paradigm consisted of point-to-point movements between different starting and target locations in which the position and orientation of the hand at the start and target location was controlled. This led to 5 possible arm postures (shoulder, elbow and wrist angle) for movements between 3 sets of starting location and targets (C, L, R in Fig 1). The reaching movements were made in horizontal plane approximately 10 cm below the subjects’ shoulder level, while the forearm was supported against gravity by an air sled. As the subjects’ torsos were immobilized with a four-point harness-style seatbelt and the forearm movements constrained to a two-dimensional plane, the upper limb can be approximated by a 3 degrees of freedom system (shoulder, elbow and wrist movements in the horizontal plane) whose configuration is uniquely specified by the experimentally controlled x and y position and the orientation of the handle. To verify whether the experiment was unaffected or only minimally affected by the un-modeled degrees of freedom of the upper limb, we also measured the elbow angle with a goniometer.

### Starting Location and Target Specification

The starting locations and targets were selected individually for each subject to fulfill experimental requirements while ensuring subject comfort. The aim was to choose postures that differed in either Cartesian, joint angle or hand orientation. Subjects placed their hand directly in front of their torso with an elbow angle around 90° and this defined the neutral posture and central hand location (C). Subjects were then asked to flex and extend their wrist maximally and the center of the range defined the neutral wrist angle (0°). Note that this neutral wrist angle differs from its anatomical definition [23] since subjects have a greater range of flexion than extension from anatomical neutrality. Starting with the hand on the central location (C) with the wrist flexed 30°, the rightward starting location (R) was defined as the location of hand when it now rotated 60° to end in 30° extension but with the arm configuration (shoulder and elbow) maintained. The leftward starting location (L) was similarly defined in the opposite direction. The targets were taken as 10 cm away from each starting location in the direction of the forearm in the neutral posture.

Using the three locations (L, C, R) 5 limb postures (Fig 1: 1–5) were defined by placing the hand on the target with different hand orientations (30° flexion for L and 30° flexion and extension for C & R; Fig 1). Contextual effects of the three coordinate systems (Cartesian, joint angles, and hand orientation) can be individually examined by picking different sets of four postures (out of five) and pairing them with CW and CCW force fields (shown by red and blue lines Fig 1). Depending on the coordinate system, each force field (indicated by the colored lines) can be dissociated based on the posture specified in Cartesian hand position (2–3 and 4–5), or shoulder-elbow joint angles (1–2 and 3–4), or hand orientation (1–3 and 2–4).

To ensure that subjects were comfortable, they made movements between the targets under a constant force whose magnitude approximates the maximum force that will be experienced during the experiment (3.75 N). If a subject reported any (wrist) pain during the movement, then the calibration was restarted from the beginning with adjusted instructions; for example, if a subject reported pain while flexing the wrist when the handle was further away, then in the next calibration the neutral hand position was chosen to be closer to the body and the subject was asked to extend more and flex less to find the neutral hand orientation. This procedure was iterated until a comfortable set of postures was found.

### Experimental Procedure

While grasping the handle of the manipulandum, subjects performed 10 cm reaching movements (Fig 1A). The hand was represented by a red cursor (circle radius 0.5 cm) and the starting and target locations of the movement by white circle (radius 1 cm). Both cursors and start and target circles also had an orientation indicator, a dark bar from the center to the edge of the circle (thickness 25% of the radius). The task involved making a reaching movement from the start location to a target location both of which had the same orientation on each trial. To start a trial subjects needed to match the position and orientation of the starting location for at least 600 ms (position and orientation tolerance 0.5 cm and 6° respectively). The corresponding target then appeared 10 cm away and subjects were required to reach the target and the movement ended with a tone when the hand was within 0.55 cm of the target and within 12° of its orientation for 750 ms. Subjects were encouraged to make the movement within 270–420 ms (and received feedback ‘great’ if they did so). If they were outside this range but within 200–520 ms they received feedback ‘good’. Outside this range they received ‘too slow’ or ‘too fast’ as appropriate. To discourage overshoot ‘overshoot’ feedback was provided if the hand moved more than 11 cm from the starting location.

Since the same extrinsic hand orientation is required at the start and the end of the trial, it is important to note that the motor skills that subjects are required to develop over the experiment, primarily involve control of the torque at the shoulder and elbow joint. It is often assumed in the previous studies on the force-field adaptation that the subjects maintain a constant wrist angle during the movement [2,24–26] and therefore the wrist joint has a negligible role in the dynamics of movement. A recent study has also shown that the wrist is unperturbed by moving in a force field (see Figure 4B of Berniker et al. [20]), which means that subjects tend to stiffen the wrist without developing any active force compensation strategy.

Each movement was either made with the vBOT motors off (null field) or with a velocity-dependent curl force field applied to the handle (force field trials). On force field trials, the force *f* depended on the hand velocity $v={\left[\dot{x},\dot{y}\right]}^{\text{T}}$:

$$f=kRv=k\left[\begin{array}{cc} 0 & -1\\ 1 & 0 \end{array}\right]\left[\begin{array}{c} \dot{x}\\ \dot{y} \end{array}\right],$$

where force constant *k* was set -0.13 N s/cm for clockwise (CW) and 0.13 N s/cm for counter clockwise (CCW) fields. Channel trials were used to assess feedforward adaptation. In a channel trial, the movement was confined to a simulated mechanical channel from the starting to central target with a spring constant of 8,000 N/m and a damping coefficient of 20 N s/cm orthogonal to the wall [27,28].

Each experiment consisted of three conditions (Cartesian, Joint angles and Hand orientation conditions), which differed in how the direction of the force field was associated with the 4 movements used in the condition. Each condition consisted of 360 trials, 60 pre-exposure trials (null field) followed by 200 exposure trials (force field) and 100 post-exposure trials (null field). Movements were performed in blocks of 20 trials, consisting of 16 field trials (4 for each configuration) and 4 channel trials. The order of trials within a block was randomly permuted with the restriction that two consecutive channel trials were not allowed. Overall, the experiment consisted of a total of 1080 trials, during which all three conditions (360 trials per each) in Fig 1 were tested.

In this study, each subject performed the 3 conditions in a counterbalanced order in the same session (four subjects each experienced one of the 6 possible orders). Force field direction was also counterbalanced across subjects so that for a particular order the assignment of field direction to movements were either as depicted in Fig 1 (two subjects) or opposite to Fig 1 (two subjects). Force field directions shown in Fig 1 were used for 12 subjects, while the directions were reversed for the other 12 subjects (i.e. red for CCW and blue for CW). Similarly, the order of the three conditions was counterbalanced across all of the subjects, with 4 subjects performing each of the six possible sequences. Importantly this means that the same limb configuration (e.g. 3) occurs with the same force field (e.g. CW) in all three conditions. It is only the overall allocation of force fields (pattern across the limb configurations) that changes between conditions. Therefore, any biomechanical effects arising from the use of five different limb configurations, such as variations in joint torques and mechanical properties of the upper limb, apply equally to the all three conditions and therefore would not affect the result. Also, any systematic or random variations in the limb configuration due to un-modeled kinematics of the limb, such as protraction and retraction of the scapula, would also affect all three conditions and will be factored out by our counterbalanced design.

### Data Analysis

Data analysis was performed in Matlab R2012a. Learning was examined by calculating the maximum perpendicular error (MPE) in the (null or force) field trials and the force compensation in channel trials. MPE was measured as the maximum deviation of the cursor from a straight line connecting the starting and target positions between the start and end of each movement. The force compensation represents the amount by which a subject specifically compensates for the predicted force field. This measure is more sensitive to learning than kinematic measures as error can be reduced by co-contraction induced changes in limb stiffness [29,30]. Force compensation *C* (in percentage) was computed on channel trials as:

$$c=\frac{\int n^{\text{T}}\;\bar{f}dt}{\int n^{\text{T}}kRvdt}\times 100,$$

where $\bar{f}$ is the measured handle force and *n* is a normal vector to the mechanical channel. The integral of force compensation was calculated from the start to the end of the movement. The values of force compensation therefore represent the percentage of full compensation for the velocity–dependent force field [11,31]. To collapse values across subjects the appropriate axis of the kinematic and force data was reversed between the subjects with the opposite force fields.

Hypothesis-based planned comparisons were performed and uncorrected *p*-values were reported to determine statistical significance. Statistical differences were assessed with an ANOVA using the general linear model in SPSS Statistics 21 (IBM), with subjects as a random factor. To evaluate learning, we defined four epochs in each condition: late pre-exposure, early exposure, late exposure, and early post-exposure, each of which consists of four trials. A general linear model was used to test if the force field produced significant changes in movement errors by comparing MPE on late pre-exposure and early exposure epochs (2 levels) with a factor of condition (3 levels). To test if the error was significantly reduced during exposure, another linear model was used to compare MPE on early exposure and late exposure epochs (2 levels) with a factor of condition (3 levels). The existence of an after-effect of learning was evaluated by another linear model comparing late pre-exposure and early post-exposure epochs with a factor of condition (3 levels). Similar tests were also performed on force compensation where appropriate. If a significant main effect was found, Tukey’s HSD (honest significant difference) post hoc test was used to examine differences. Statistical significance was considered at the p < 0.05 level for all tests.

## Results

Subjects performed movements in 5 different arm postures. We first examined the arm configuration (shoulder and joint angles) between adjacent configurations. ANOVAs were performed on the goniometer data to check whether there were statistical differences in elbow angles among movements with same arm configuration (1–2 and 3–4) and those with different arm configurations (2–3 and 4–5). The result, summarized in Table 1, shows that there were no significant differences in elbow angles between same configuration movement (movement 1–2 and 3–4) in initial angles and movement 1–2 in final angles. There was a marginally significant difference in only the final angles of the movement pair 3–4, but their mean difference was very small at 1.65°. On the contrary, elbow angles in non-adjacent movements were significantly different both in initial and final angles. The durations of movements in the channel trials (mean 0.293 ± se 0.0240 sec across subjects) were kept consistent across the five possible trajectories (F_4,5179_ = 0.854; p = 0.491).

**Table 1:** Elbow angles.

	Movement pair	Initial angle difference	Final angle difference
Same arm configuration	1 and 2	0.512°	0.390°
		F_1,23_ = 0.329; p = 0.572	F_1,23_ = 2.366; p = 0.138
	3 and 4	0.471°	1.65°
		F_1,23_ = 2.523; p = 0.126	F_1,23_ = 6.350; p = 0.019
Different arm configurations	2 and 3	14.3°	14.4°
		F_1,23_ = 146.159; p < 0.001	F_1,23_ = 89.956; p < 0.001
	4 and 5	15.8°	16.0°
		F_1,23_ = 108.591; p < 0.001	F_1,23_ = 71.503; p < 0.001

Differences in elbow angles (means) and result of ANOVAs testing statistical differences between elbow angles in adjacent movements

**Fig 2:**
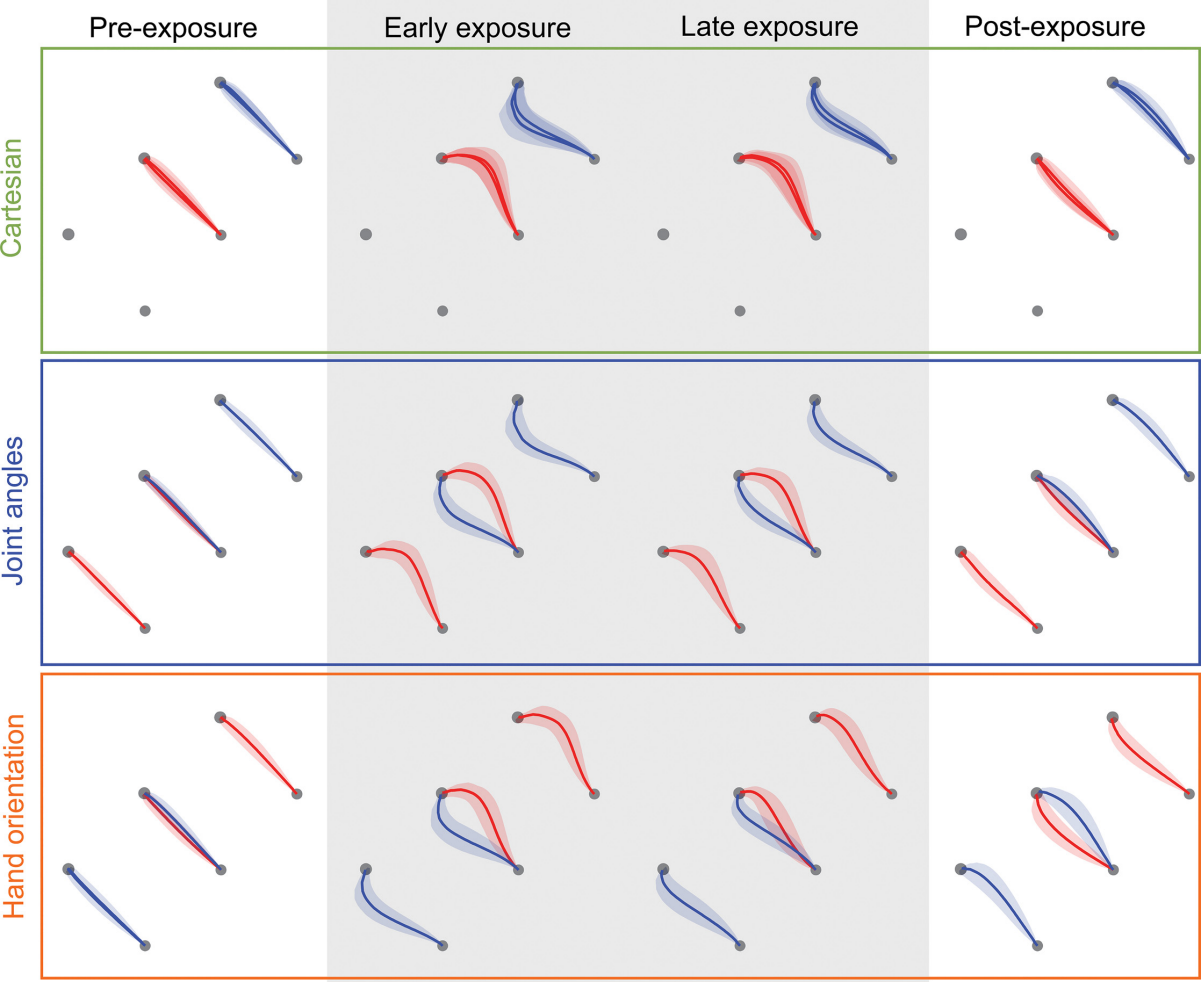
Trajectories. The mean (solid line) and standard deviation (shaded region) across subjects of hand paths is shown for the pre-exposure, initial exposure, final exposure, and post-exposure epochs of the experiment in each of the three experimental conditions. The paths have been flipped around the inter-target line for the half of the subjects that had the force field directions reversed.

Subjects made relatively straight movements during the pre-exposure trials (Fig 2). Once the force fields were introduced, the trajectories showed large deviations from a straight line, in the direction of the applied force (rightward for clockwise and leftward for counter-clockwise force field). In the late period of exposure trials, the deviations were reduced in all conditions. In the post-exposure period, the movements in the hand orientation condition strongly deviated in the opposite direction to the initial errors, indicating substantial after-effects of the learning. In contrast, in the other two conditions the initial post-exposure trajectories were close to the straight line. This suggests that in these two conditions there was less predictive force compensation to the force fields. Together, these findings indicate that greatest reduction in interference occurred in the hand orientation condition.

**Fig 3:**
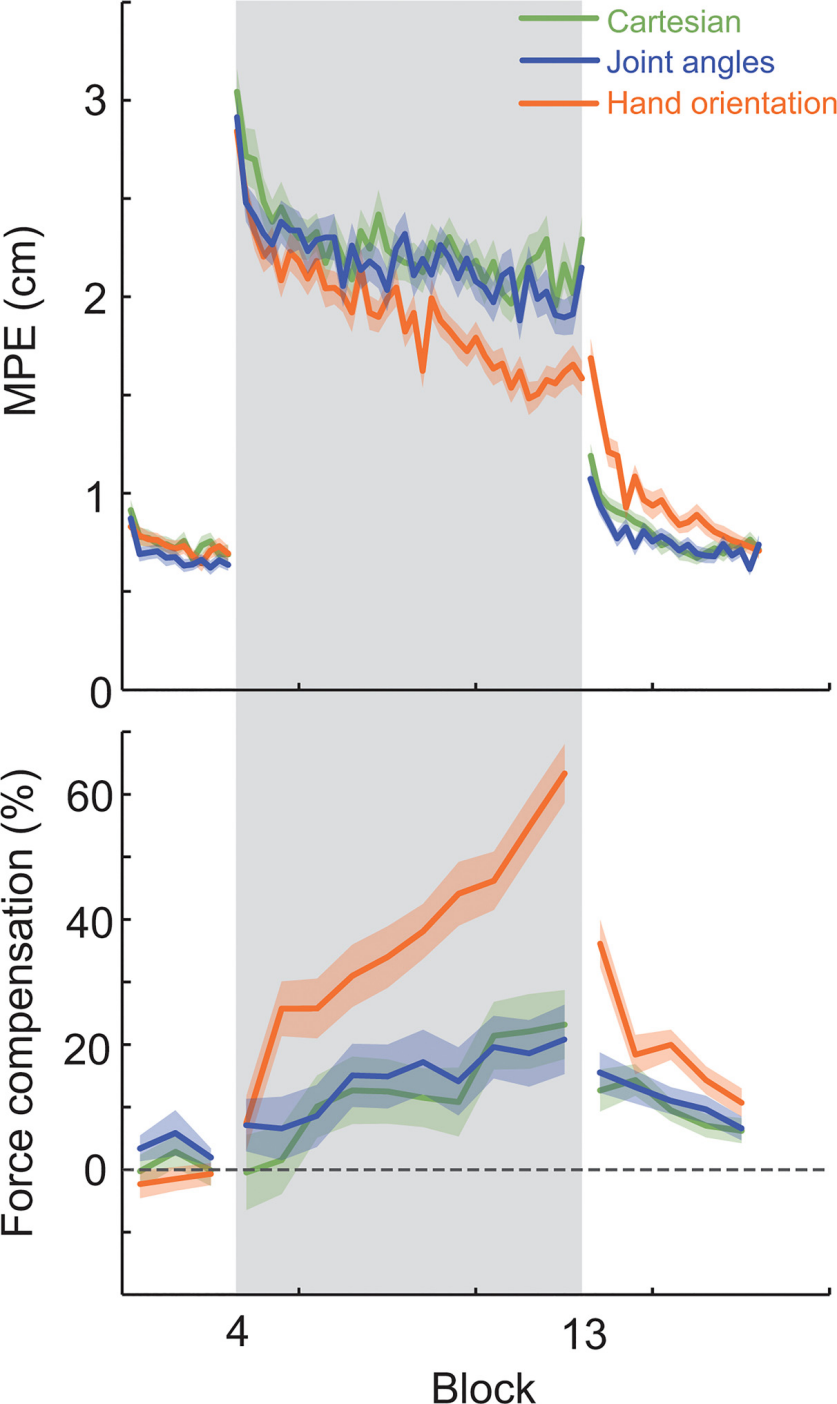
Learning. Top: the mean (solid line) and standard error of the mean across subjects (shaded region) of the maximum perpendicular error throughout the experiment. The grey shaded region indicates the period over which the curl force fields were applied. Bottom: the force compensation.

To quantify these observations, the maximum perpendicular error (MPE) and force compensation were calculated across all the experimental trials (Fig 3). The MPE which was close to zero in the pre-exposure epoch, was dramatically increased during the initial exposure epoch (F_1,23_ = 455.355; p < 0.001), with no significant differences among the force fields types (F_2,46_ = 0.360; p = 0.699). To confirm that there was no difference in the peak MPE during the initial exposure trials, an ANOVA was run on the first 4 exposure trials in the three force field conditions, demonstrating no significant main effect (F_2,46_ = 0.516; p = 0.600). For all three conditions, the large errors at the beginning of the exposure consistently dropped during early exposure (block 4). However, the force compensation in this same period demonstrates distinctive differences among conditions: subjects compensated for 20–25% of the force field in the hand orientation condition, but less than 10% for the other two conditions.

This distinction continues during the middle and late exposure trials (blocks 5–13). While the MPE exhibited little reduction in the Cartesian hand position and joint angle conditions, it significantly decreased in hand orientation condition. To examine the degree of adaptation, we used an ANOVA to contrast the MPE in early exposure to the late exposure for all three force fields with main factors of force field condition (3 levels) and epoch (2 levels: early and late exposure), with random effect of subjects. There was a significant main effect of epoch (F_1,23_ = 145.292; p < 0.001) demonstrating significant learning across the three conditions, but also a main effect of condition (F_2,46_ = 9.483; p < 0.001) indicating that there were differences in the amount of error reduction in the three field types. A post-hoc test demonstrated the largest decrease in hand orientation trials (p = 0.003) with no differences between the Cartesian and joint angles conditions (p = 0.518).

In accordance with the MPE data, subjects in the hand orientation condition gradually increased their force compensation, reaching 60% by the late exposure period. In contrast, only a small increase in the force compensation (~20%) was observed in the other two conditions. The relative changes in force compensation were further examined with an ANOVA with main factors of force field condition (3 levels) and epoch (2 levels: pre-exposure and late exposure) and random effects of subjects. There was a significant main effect of epoch (F_1,23_ = 142.365; p < 0.001) demonstrating significant learning across all three conditions, but also a main effect of condition (F_2,46_ = 28.556; p < 0.001) indicating significant differences in the final degree of compensation across the three conditions. A post-hoc test indicated that the largest increase occurred for the hand orientation condition (p < 0.001) with no difference between the Cartesian and joint angles conditions (p = 0.964).

This difference between the compensation in the three conditions was also found during the initial post-exposure trials, with the largest after effects seen in the hand orientation condition (Fig 3). An ANOVA contrasting the MPE in early post-exposure to the late pre-exposure for all three force fields (main factors of force field condition (3 levels) and epoch (2 levels), and random effect of subjects) found a significant main effect of epoch (F_1,23_ = 121.759; p < 0.001) demonstrating significant after-effects across the three conditions, but also a main effect of condition (F_2,46_ = 13.968; p < 0.001) indicating differences in the size of after-effect in the three field conditions. A post-hoc test found the largest after-effects in the hand orientation condition (p < 0.001) with no difference between the Cartesian and joint angle conditions (p = 0.369). Similar results were obtained from an ANOVA contrasting the force compensation in early post-exposure among all three force fields (main factor of force field condition (3 levels) and random effect of subjects). After a significant main effect of condition (F_2,46_ = 23.557; p < 0.001), a post-hoc test showed the hand orientation condition had the largest force compensation (p < 0.001), with no significant difference among the other two conditions (p = 0.968).

## Discussion

Subjects were presented with opposing force fields during reaching movements where the direction of the force field was uniquely separable in different sessions only by 1) Cartesian hand position, 2) shoulder and elbow joint angle or 3) hand orientation. While subjects in all conditions showed some adaptation to the force fields, the greatest adaptation was observed in the hand orientation condition (~60%) with the amount of adaptation about three times larger than either of the other two coordinate systems (~20%). The results demonstrate that differences in hand orientation allow subjects to separately learn two opposing force fields efficiently. With the other two conditions, Cartesian hand position and joint angle changes, subjects were able to learn the two fields only slowly.

Although there were large initial decreases in the kinematic error in all three conditions, the increase in predictive force compensation for the hand orientation condition was substantial compared to the other two conditions. This suggests that different strategies were used to reduce the errors in early exposure period for the three conditions. For Cartesian and joint angle conditions, subjects may have used co-contraction [29,32] or increased feedback gains [33,34] to reduce the errors, in contrast to the hand orientation condition in which they developed a feedforward force compensation strategy as can be seen by the increased predictive force on the channel trials [11,31,35].

Our result extends previous studies on the reduction in interference to dynamic learning [5,14,17] by carefully controlling the coordinate system of the spatial separation. Results of these previous studies were usually interpreted, without any experimental evidence, as a contextual effects arising from the separation in joint angle coordinates. While the assumption is based on results of classical studies suggesting that motor memory is encoded in intrinsic, joint-based coordinates [2,24–26], recent studies [20,36] suggested that the generalization pattern of the arm dynamics can not explained in a single coordinate system, *e*.*g*. joint based coordinate, and can be better explained by a mixed representation of joint angles, Cartesian and hand orientation coordinate systems. This implies that the interference reduction observed in the previous studies could have resulted from a separation in either of the other two coordinate systems, or mixture of all three. Our study evaluates the efficacies of the contextual information from each individual coordinate system in reducing interference, and shows that for the spatial separation of the hand similar to previous studies [17] that the hand orientation coordinate system plays a privileged role.

One mechanism by which hand orientation may be privileged is that changes in wrist orientation may lead to a large change in perceived orientation in intrinsic space, that is changes of the proprioceptive input. Compared to small changes in endpoint location, the different hand orientations may be more separated in sensory space than the differences in, for example, joint angles. However, despite the larger angle change for the wrist compares to shoulder and elbow in our experiment it is known that proprioception is more sensitive in proximal joints, which will tend to counteract the sensory difference. In our experiment the change of wrist angle was 60° whereas those of elbow and shoulder angle were both less than 15°, resulting in around a four times larger difference in angular space. Based on Tan et al. [37], the just noticeable differences for wrist, elbow and shoulder joint angles differ by maximum 2.5 times (2.0° for wrist and 0.8° for shoulder), suggesting that the proprioceptive difference of the wrist posture in sensory space may be still bigger (approximately 1.6 times larger) than that of elbow and shoulder postures used in our experiment. It is open question whether such an increase in sensory space can account for the large difference in force adaptation seen in our experiments (approximately 3 times larger) between the hand orientation and joint angle conditions. Nevertheless, when considering learning two opposing force fields separated by 7 cm our results suggest that changes in hand orientation has the greatest effect compared to changes in only joint or Cartesian coordinates.

We can directly compare our results to Hwang et al. [17]. In both their study and ours the separation in hand space was similar for the two force fields (6.3 ± 0.9 cm for our experiment and 7 cm for Hwang et al. [17]. In their study they found substantial learning (although not quantified with channel trials) with a performance index of around 50%. However, in their study, this separation occurred in all three coordinate systems. When we examined this offset in only Cartesian coordinates with the other coordinates uninformative then learning was modest at around 20%. A similar amount of adaption occurred when this separation occurred only in joint space. In contrast, changes in hand orientation led to substantially more learning. We suggest that this indicates that most of the improvement in performance found by Hwang et al. [17], arose from the difference in hand orientation (although their change in hand orientation was likely smaller than in our study), but that all three likely contributed in order to maximize the sensory differences between the two conditions. We speculate that the sensorimotor system may try to cluster high dimensional proprioceptive inputs so as to map them to different motor outputs. As the largest proprioceptive difference arises from the wrist in our experiment then this will dominate any clustering and lead to reduced interference primarily in the hand orientation condition. Ideally we could examine similar sized changes in the proprioceptive space for joint and hand, but the limb geometry limits the size of possible joint changes in the plane with the same Cartesian hand location.

Previous studies have also shown a strong contextual effect related to the orientation of grasped objects. Ingram et al. [19] demonstrated that the motor system maintains multiple representations of the dynamics, each of which are specified by a specific grasp, *i*.*e*., the orientation of hand. In a following study [38] they showed that subjects are able to immediately recall the grasp-dependent dynamics from the visual orientation of the tool. These results suggest that strong contextual effect of the hand orientation observed in our experiment is possibly due to an inherent mechanism of prioritizing the hand configuration, which enables dexterous object manipulation.

There are many neurophysiological studies examining the coordinate frames in the motor cortex involved in reaching with divergent conclusions [39]. Neural tuning in the motor cortex has been associated with both Cartesian kinematics [40–42], and joint or muscle based intrinsic coordinates [43–45]. Moreover, one study found evidence supporting both movement (Cartesian-like) and muscle based (intrinsic-like) tuning [46]. These variations in neural tuning might explain the flexibility in the manner in which dynamics appear to be encoded [20,47]. Although some studies have examined the neural firing during tool use [48,49], no work has yet investigated whether the neural activation in motor cortex exhibits tool-based tuning. Our work demonstrating that hand orientation has a privileged role in the reduction of motor interference suggests their may also exists neurons with object-based tuning that could be contributing to the differences in the learning.

In conclusion, we have shown that, among three coordinate representations tested, hand orientation works as the most effective contextual cue that allows subject to separately learn two motor skills. The result leads to an interpretation that our sensorimotor control system uses hand orientation as a privileged contextual cue to encode motor memories.
